# Menstrual health and period poverty in Lebanon during economic crisis: A qualitative analysis of the challenges and recommendations

**DOI:** 10.3389/frph.2022.920461

**Published:** 2022-08-26

**Authors:** Malika Elhage Hassan, George Doumat, Darine Daher, Abdul Hafiz Al Tannir, Bashar Hassan, Charbel Chidiac, Hussein Hariri, Taha Hatab, Alaa Abou Daher, Omar Ezzedin, Fouad M. Fouad

**Affiliations:** ^1^Faculty of Medicine, American University of Beirut, Beirut, Lebanon; ^2^Department of Epidemiology and Population Health, American University of Beirut, Beirut, Lebanon

**Keywords:** menstrual health, menstrual health management, economic crisis, period poverty, Lebanon, women's health, reproductive health rights, human rights

## Abstract

**Objective:**

Recently, severe period poverty has had a dramatic spread throughout Lebanon as a result of several crises: the COVID-19 pandemic, the Beirut explosion, and the economic collapse. Period poverty is the lack of access to menstrual hygiene materials, comfortable environments, and adequate education about menstrual health. Due to the great implications of period poverty on Lebanese women's health, our study aims to explore stakeholder's perspective on the Lebanese public health policy regarding menstrual health, the evolving challenges it faces in the context of the current economic collapse, and to suggest recommendations for solutions.

**Methods:**

Our study is qualitative in nature, where data collection was done *via* online semi-structured interviews with stakeholders from the public and private sectors of the Lebanese healthcare system in addition to non-governmental organizations (NGOs) and physicians. Data were then analyzed based on themes and subthemes that emerged from the interviews.

**Results:**

Nine stakeholders were interviewed: five from NGOs, two obstetrics and gynecology physicians, and two public sector representatives. The challenges to menstrual health were subcategorized into previously existing and new ones. The consequences of poor menstrual health were tackled on the mental, physical, and social levels. Stakeholders suggested both short-term and long-term recommendations. Short-term recommendations included decreasing the monetary burden by subsidizing menstrual products or *via* a coupon system. Long-term recommendations included proper education on multiple levels, cooperation between key players in the private and public sectors, and encouragement of local production to ensure future sustainability.

**Conclusion:**

Menstrual health is a neglected public health issue in Lebanon, causing detrimental effects on girls and women residing in the country. Proper planning and collaboration between the private and public sectors are required to address this human rights issue.

## Introduction

Menstruation is a biological process that is charged with social and economic significance (1). The World Health Organization 1948 constitution defines health as the “state of complete physical, mental, and social wellbeing and not merely the absence of disease or infirmity” (2). In recent efforts to improve equitable women's health, menstrual health (MH) has been receiving more importance in public health policy (3). Effective MH, as defined by the Global menstrual Collective, is not merely the absence of disease, but it also entails a state of complete physical, mental, and social welfare, in relation to menstruation (4). Adequate and hygienic MH is a human rights issue and should be treated as such (5).

Ineffective MH has implications across health, education, work, and social status (6). Furthermore, the availability of appropriate products and adequate menstrual hygiene settings are unequal across various socioeconomic and geographical levels, further complicating MH (3).

Numerous publications and recent international conferences highlight how MH is a crucial yet often neglected dimension of global sanitation, which is further amplified in low and low-middle-income countries (7). Every day, an estimated 200 million menstruating girls and women in low-income countries suffer from the lack of access to adequate sanitary products (8). High costs and decreased availability lead to the decreased use of disposable and reusable sanitary napkins/pads, further contributing to the problem in low and low-middle-income countries (9).

Since 2019, Lebanon has been dealing with a worsening economic collapse and crumbling banking sector, leading to hyperinflation and the Lebanese currency losing more than 93% of its value (10). Subsidization efforts of essential goods like fuel and medications failed to include menstrual products (11). Sanitary pads and other menstrual products now cost at least four to 10 times the original prices before the financial downturn (11). The situation forced women to seek alternatives, either by compromising quality and using cheaper brands or by repurposing other products, like diapers and rugs (12).

Problems with MH are not new in Lebanon; however, they have been previously mostly confined to individuals from lower socioeconomic backgrounds, such as refugees (13). The current economic collapse has caused period poverty to become a concern to women and girls all over the country. A recent survey in Lebanon showed that 76.5% of women have difficulty accessing menstrual products (13). Among the surveyed women, 57.6% report using the same pad for longer, while 56.9% of women are opting for cheaper alternatives (13). Poor MH among women and girls in Lebanon during the crisis has demonstrated significant repercussions: 43% report anxiety and stress about MH, 36% suffer from physical symptoms and 35.9% avoid engaging in daily activities due to poor period management (13). Inadequate MH nowadays is also leading to psychological and social problems. Moreover, 46.5% of the surveyed women report feeling ashamed of their periods and 47% of the participants are afraid that other people would know that they are menstruating (13).

In this project, we aim to explore the Lebanese public health policy regarding menstrual health in the context of the current economic collapse. Specifically, we will address the lack of policies and identify recommendations aimed at accessing menstrual health materials, education, and dealing with potential outcomes in the education and labor sectors.

## Materials and methodology

### Study design

Our study is a qualitative study where data collection was done *via* semi-structured questions with nine stakeholders. The methodological approach used is the framework method of interpretive research (14). This study is a primary descriptive study exploring the topic using open-ended interview questions and drawing conclusions based on findings.

Participants' selection was purposive. Interviewees were chosen to encompass the public and private sectors of the Lebanese healthcare system, in addition to non-governmental organizations involved with women's health, particularly menstrual health. An interview guide with four main questions was developed in English since all interviewees can understand and express themselves in English. Additional probing questions were added as follow-up based on the interviewee's role and area of expertise (Table 1).

**Table 1:** Themes, subthemes, and corresponding guiding questions.

**Theme**	**Subtheme**	**Guiding questions**
Challenges	General	In your opinion, what have been the main persistent challenges facing women's menstrual health in general, in Lebanon, even predating the current economic crisis?
	Marginalized groups	What are additional challenges faced by marginalized communities, such as refugees and domestic workers?
Economic	Budget	Is there a budget/resources allocated for women's health?
	Economic crisis	How do you think the current economic crisis has exacerbated the current problems facing women's menstrual health in Lebanon?
Medical	Type of complaints	What are the most common complaints you typically see in your practice/clinic regarding this topic?
	Frequency of complaints	How has it affected the frequency of visits and have you seen an increase in menstrual health related complaints?
Consequences	Consequences	What do you think are the main consequences that arise from the current problems regarding women's menstrual health in Lebanon?
Action	Organizational level	What was the focus of your work regarding menstrual health management before the economic crisis? How has it changed as a result of the crisis? Do you feel that the economic crisis has affected your ability to perform the goals of your initiatives?
	Governmental level	Why haven't menstrual health products been subsidized after the onset of the crisis, given their importance and impact on women's health? Given that for example, men's razors are subsidized? Who makes the decisions of what products to subsidize? Have other bodies requested for these products to be subsidized?
	Key players	Who are the players that you think can most effectively cause change?
Recommendations	Short term	What are your short term recommendations on solving this issue?

All interviews, except one, were conducted *via* online Zoom/Webex meetings as per the interviewees' preference. The one in-person interview was conducted in-office. Oral consent was obtained at the beginning of each meeting. Two medical students conducted each interview, both male and female. One student asked the questions, while the other typed the responses to ensure a smooth, uninterrupted flow of the interview. No one other than the interviewers and interviewees was present during the interview process. The duration of the meetings averaged around 20 min. The interviews were conducted from August to September 2021. No changes or modifications were made to the transcripts after the interviews. Participants were not provided the analysis for feedback or editing.

### Recruited stakeholders

Recruitment of the following stakeholders was based on their expertise and experience in menstrual health management in Lebanon, be it through work in the public sector or at a ministry level, physicians practicing as obstetrics and gynecology (OBGYN) specialists, or in non-governmental organizations (NGOs) dealing with women's health.

The participants were approached *via* email and were told the subject matter of the research and asked if they were interested in participation before the interviews were conducted.

Oral consent was obtained at the beginning of every interview and approval to record their answers in writing was secured. The interviewees could withdraw at any point during the interview. They also received a copy of the question guide by email prior to the interview.

### Data analysis

Data were collected through the verbatim transcription of the interviews. Microsoft Excel was used to tabulate the responses per the questions asked. The interviewees' answers were tabulated in separate columns. Framework analysis was used to analyze the results based on themes (14). A deductive approach (theory-driven) was used to organize the data into four main categories, following the organization of our questionnaire. Afterward, an inductive approach (data-driven) was implemented to add categories based on quotes that did not fit the data-driven categories. The coding process identified concepts based on existence, meaning the concept would be counted if it appeared at least once. Major and minor themes were derived for analysis that was consequently summarized.

## Results

### Current menstrual health policy

No current policy on menstrual health in Lebanon was found to exist.

### Pre-existing challenges

In responding to the main challenges facing women's menstrual health in Lebanon that predated the current economic crisis, respondents stated that menstrual cycle knowledge is very limited, there is a lot of confusion regarding what a normal menstrual pattern vs. an abnormal one is, and what is related to fertility vs. what is not.

“Period poverty is the lack of access to menstrual products, a bathroom where women can change, and a safe place where women can freely talk about their issues.”—Local NGO cofounder.

As for MH challenges affecting marginalized communities, such as refugees and domestic workers, interviewees highlighted poor living conditions, lack of access to proper infrastructure within camps, and low salaries. Possible solutions to mitigate these challenges are awareness campaigns offering boxes with tissues, pads, hygienic lotions, and soap designed by the UNFPA. According to a local NGO, reports showed that girls within these marginalized communities did not know what the kit was used for, and they wanted cosmetics instead, which highlights the “menstrual knowledge” challenge being at the core of the problem. Although dignity kits are not distributed anymore because of their cost, awareness campaigns are still being held by the UNFPA.

### Emerging challenges related to the economic crisis

The stakeholders described women's menstrual health in Lebanon in the context of the current economic crisis as a disaster. Due to the exponentially increasing expenses of pads, women are resorting to makeshift ways of handling their periods like old clothes or cotton and nylon coverings. Several initiatives and organizations had shifted their efforts toward focusing on the distribution of MH kits, rather than awareness campaigns.

“Women right now do not care about learning; they just want their products so that they can survive in their daily life.”—Founder of another local NGO.

Another problem is the government's lack of funding and support toward women's products even though other sanitary products, like razor blades, are supported. Thus, the financial decisions can be described as “patriarchal.” The respondent from the Ministry of Public Health stated that there was no government budget allocated for women's health. The respondent recommended the focus be shifted toward getting the support of NGOs and the UN, seeing how the government is overwhelmed during this critical time dealing with the lack of access to medications and vaccines in the country.

Moreover, there is only one domestic producer of sanitary products in Lebanon, with the rest being imported. Additionally, only five out of 15 raw materials needed to produce sanitary products are subsidized in Lebanon. Hence, locally produced sanitary products are currently not cheaper than their imported counterparts for domestic use. Another emerging challenge addressed was water scarcity. So, even if the issue of educating women on reusable pads and menstrual cups is addressed, the lack of proper access to water, soap, and adequate temperatures for washing renders these alternatives ineffective and potentially dangerous due to an increased risk of infections.

### Main consequences identified

Furthermore, when asked about the various consequences arising from poor MHM in Lebanon, most of the responders believed that what women are currently experiencing is a combination of physical, social, and mental challenges.

Responders considered mental and social consequences as the most overwhelming, specifically the humiliation women are suffering from on daily basis. Women and girls are experiencing significant distress and anxiety, negatively impacting their social life. Period poverty is hindering women's empowerment, as many tend to conceal psychological outcomes and not seek medical support. Women are also being less productive: missing school/workdays and trapping themselves throughout menses.

Regarding physical health, there was an increase in skin and genital infections from resorting to makeshift and sub-optimal methods during menses. Some of the reported conditions include perineal dermatitis, sexually transmitted infections, and dermatopathies. Most patients delay seeking treatment, resulting in significant medical morbidities which are also greatly attributed to lack of education. In addition to this, the restriction on mobility female experience, as a consequence of poor menstrual management, amplifies the social consequences mentioned before.

### Stakeholder's recommendations for action

After exploring the various outcomes and consequences associated with poor MH, interviewees were asked about possible solutions and recommendations. All responders agreed that immediate measures must be taken to alleviate the burden on women.

Regarding short-term recommendations, the interviewees suggested providing and advertising free healthcare clinics and gathering donations to offer disposable pads or washable pads which are more cost-effective. Additionally, it was suggested that local production companies decrease their exports to increase local availability. Another proposed solution is a voucher system that can guarantee access to sanitary products exclusively, which will ensure fair distribution by removing money from the equation. Furthermore, a stakeholder suggested creating a chain that links the Center for Social Services, primary care centers, NGOs, municipalities, and schools to regulate the acquisition and distribution of pads, particularly to those who need them the most.

Moving on to long-term solutions, most responders suggested education and issuing governmental policies as the main pillars. To tackle the lack of education, the Ministry of Higher Education must implement menstrual health education as a fixed part of middle school curricula which aids in raising awareness among both males and females, thus normalizing and de-stigmatizing the topic. This school education programs should be comprehensive and aimed at girls, boys, and teachers because everyone has a role to play in solving the problem. These formal education programs can be supplemented by independent awareness campaigns organized by local NGOs. Some stakeholders believed that environmentally sustainable menstrual products are the future; however, others warned that the current infrastructure and stigma in Lebanon prohibit this. For example, some unmarried women and girls may fear that menstrual cups may compromise their virginity and are thus taboo to use. It was stressed that high levels of advocacy by the public are required to cause change, and this can only be done with a high level of public awareness. Interviewees also believed that local production needs to be increased substantially and to that end, policy and legislative amendments need to be undertaken. This can be best achieved by conducting feasibility studies with the Investment and Development Authority in Lebanon (IDAL) which proposes solutions such as offering fast-track construction permits, reduction in land registration fees, and tax exemption on raw materials to interested investors. Other solutions included diverting the production of some factories, like diaper-producing factories, into sanitary pad production.

There was a consensus among responders that the cooperation of many parties is necessary to cause change, and everyone particularly highlighted the government's role. The ministries of industry, health, and education were mentioned. The role of the ministry of the industry includes subsidizing raw materials for the local production of menstrual hygiene products and removing taxes on finished products. They believed that the government should play an active role because “pads are not a luxury like razors or shaving creams”—they should be available and affordable to all. Moreover, the government could be the key to organizing efforts coming from different sources under one umbrella that will maximize impact. Combining the organization of a governmental body, funding of the private sector, expertise of the healthcare professionals, and initiative of the NGOs, will create a clear plan to solve the problem.

## Discussion

Experiencing menstruation safely and cleanly is a human rights issue (5). In Lebanon, attaining proper MH has always been a struggle, especially in marginalized communities. The three crises, namely, the devaluation of the Lebanese Lira, the COVID-19 pandemic, and the August 4th Beirut explosion have made period poverty a more widespread problem.

Before the economic crisis, lack of education and cultural taboos were cited as the main source of MH mismanagement. Additional challenges faced by more vulnerable populations, like domestic workers and refugees, include financial issues, poor infrastructure, and the lack of private and safe places. A 2018 report by Concern Worldwide showed that many refugees used baby diapers or tissue papers during their menses (15). The deteriorating situation in Lebanon has multiplied the number of women facing MH-related problems.

A wave of crises hit Lebanon almost simultaneously; starting with the October 17, 2019 revolution, followed by the globally devastating COVID-19 pandemic in 2020, and finally the Beirut port blast of August 4, 2020. These factors led to the devaluation of the Lebanese Lira by more than 90% (10), which is still on a downhill spiral. Consequently, problems arose in every sector and industry, including MH.

According to our responders, three main challenges became clear in the aftermath. First, as the price of menstrual pads skyrocketed (now costing more than 10 times its pre-crisis price), most Lebanese women started using fewer pads and other MH-related products (11). This was reflected in a study conducted by Plan International Lebanon which showed that 79.2% of women changed their consumption habits of menstrual health products, either by using the same pad for a long time or using cheaper alternatives (15). Some women resort to alternative measures such as “using old clothes, reusable cotton pads, and even newspapers.” These methods are used by women and girls in resource-poor countries such as Kenya, Northern Ethiopia, Pakistan, and Uganda among others (9, 16, 17).

Second, because of these makeshift methods, the interviewed physicians are reporting an increase in clinic visits regarding previously “rare” and “not-often seen” diseases, such as advanced-stage vulvar dermatitis. This is in concordance with the literature, as a study conducted by Majed, R. showed that poor menstrual hygiene was associated with an increased risk of infections and morbidity (18). Similarly, a study in Ethiopia used a self-administered questionnaire to identify women with a high risk of reproductive tract infections (RTIs) (19). Women who changed blood-absorbent material and washed their genital area only once per day were associated with higher rates of reproductive tract infections (19). Another study in India reports an increase in RTIs among women adopting unhygienic menstrual management methods, with Candida and Bacterial vaginosis being the most common infections (20).

Finally, responders agree that cultural taboo and lack of education act as the scaffold for the exacerbation of these difficulties during the current economic crisis. During the collapse of the Lebanese currency, the Lebanese government subsidized men's health products like shaving creams and razor blades, while women's health products, such as menstrual pads, were not (21). The government even subsidized snacking nuts, but not menstrual pads (22). This is just one example of the Lebanese society's dismissal of women's health, and consequently, women's rights. This leads to a variety of consequences in the lives of women in Lebanon. Interestingly, there are no publications on this type of discrimination in Lebanon and no articles were described Lebanon as a “patriarchal society,” as described by a respondent. Further investigations about this are warranted. According to the results, poor MH has physical, social, and mental repercussions. In terms of mental and social outcomes, the results indicate that females are suffering from humiliation, anxiety, and distress; they are less productive and missing school/workdays. This is in accordance with the study conducted in Uganda by Miiro et al. in 2018 which found that 20% of female adolescents report missing at least 1 or more school days each month due to menses, in addition to experiencing substantial feelings of embarrassment (23). To alleviate the burden of the embarrassment and distress that women have already gone through, it may be beneficial to create support groups for girls and women who have faced difficulties or have been bullied because they were menstruating. These would provide affected women and girls with a safe space to share and, in turn, would help them better cope with their experiences and allow them a chance to restructure their perspective about the natural process their body experiences.

The proposed long-term solutions focused on effecting change at multiple societal levels. The focus of these changes would include but are not limited to government, schools, home, and the workplace. Many responders replied that long-term solutions are in the hands of the government; however, a powerful change in societal discourse and behavior can also influence policymakers. The main culprit is, at its root, societal values and stigma. A way of changing society is through education. Without it, myths perpetuate and stigma strengthens. This is consistent with the literature as a study by Sommer et al. described that the lack of proper menstrual health education for both boys and girls was associated with exacerbated cultural stigma about menstruation (24). Education, especially in schools, will help shift the narrative surrounding menstruation from its stigma and associated myths to something much more positive and inclusive. The Ministry of Education can work on implementing updated curricula to include MH, especially at the middle school level for all children. At the level of the community, NGOs, and civil society have a huge important role to play when it comes to educating the public regarding menstrual health. Nation-wide campaigns aimed toward de-stigmatizing periods are key. It can also serve as advertising for relevant NGOs that are currently working on acquiring and dispersing MH kits in underserved communities to acquire donations for their cause. Another potential solution is the creation of dedicated public affordable/free clinics sponsored by the MoPH for women's health. These clinics could act as hubs for providing medical care but also places where underprivileged girls and women can go receive MH materials and relevant education. Additionally, NGOs can also work hand-in-hand with the government in an attempt to secure appropriate alternatives to disposable menstrual pads and tampons, seeing as the price of these products continues to skyrocket. It is not just important to educate about menstrual health in isolation, women's rights as a general concept should be bolstered in education. Empowering women to be in decision-making positions would allow for women's issues to be addressed in a more knowledgeable fashion. This in turn would encompass fixing the gaps in menstrual health and a variety of other overlooked issues regarding women's health. This is exemplified by Sommer's observation that the WASH community, which was dominated by men, did not know how to handle menstrual health management and consequently did not prioritize it (24).

Another theme addressed in the long-term recommendations is that of sustainability. There is a debate among respondents about finding alternatives for disposable menstrual pads vs. making them more accessible. On the one hand, making disposable pads more accessible requires financial and industrial frameworks to be placed to boost local production, subsidize, or cheapen the products. The results show that currently, local production is not a sustainable choice as most raw materials for menstrual pads are not subsidized. This is consistent with findings that show that local production is not necessarily always a cheaper alternative. For example, homemade pad-producing machines in India cost at least 723 GBP, without the cost of materials (25). Though Japan has begun to fund pad production in the Lebanese city of Tripoli, it is unsustainable to depend solely on foreign aid (26). Hence, subsidizing and lowering taxes on imported products and raw materials for local production in Lebanon may make these products much more affordable to consumers and may play an important role in rejuvenating, even if to a small extent, the local industries that work directly or indirectly in manufacturing them. On the other hand, it is arguable that Lebanon in its current state cannot financially support businesses by subsidizing raw materials for disposable pads or finished products, as there is concern whether subsidization, as a band-aid solution for the crisis, will even continue to exist. Hence, the proposed solution is to encourage the use of alternative products. Regarding the cost of various menstrual hygiene products, UNICEF calculated in 2019 that disposable pads cost 0.10–0.30 USD per pad with an accumulating cost for replenishment; reusable pads come at a higher initial cost of 1.50–3 USD per pad but are cheaper long term due to lower replenishment requirements; and menstrual cups have a high initial investment cost of 10 to 40 USD as a one-time purchase without recurring costs (27). Even at the lower rates that the UNFPA provides these products, there are still other non-fiscal considerations (28). The issue with alternative products is that not all women may be comfortable with them or know how to use them. Cultural context is an important consideration when advocating for certain alternatives, such as the menstrual cup. Lebanon's culture is intertwined with religious values and the concept of virginity is highly regarded in many communities. A device such as the menstrual cup may be seen as taboo due to its application inside the vagina, thereby compromising the currently held beliefs about virginity (28). Therefore, it may not be the right choice to offer religious unmarried women or girls in certain communities. Moreover, a pilot study in Uganda supporting the use of menstrual cups showed that uptake was 87% among participants. Women reported satisfactory experiences while using the cup and that they needed 2–6 months to get accustomed to it (29). Another population that requires special consideration is refugees in Lebanon. Access to clean water is shamefully limited in refugee camps—if these women were given washable pads, the water they use to wash them would not clean them (13). More information about the water quality and quantity requirements of different menstrual hygiene products is necessary to tailor recommendations for resource-poor populations. Nonetheless, a UNEP report identified menstrual cups as the most eco-friendly menstrual product. However, this report did not outline the associated water need for the cups (30). Therefore, a one-fits-all solution regarding disposable pad substitutes is not pragmatic and context should be heavily considered for many sub-populations of women in Lebanon. Solutions should include giving women the tools to make informed decisions through education and empowering them by giving them options, instead of enforcing a solution that they may not understand or be comfortable with. In this way, their rights can be protected.

## Limitations

Our stakeholders were limited in number. We managed to interview nine individuals whose roles included NGO representatives, and OBGYN doctors, with only two representing governmental authorities—one parliament member and one MoPH representative. However, no representatives from relevant manufacturing industries were interviewed, which would have offered valuable insight. Also, all interviews, except one, were done *via* WebEx/Zoom online meetings which might have limited some interviewees' ability to express themselves. Another limitation of this study is that it explored the views of a few policy stakeholders without including firsthand insight from women in Lebanon who are experiencing period poverty. More studies are needed to explore the impact of the unmet menstrual needs of women and girls in Lebanon. These studies should also include a molecular investigation to quantify the medical risks to support the observations of the interviewers in this study. Additionally, more studies are needed to test the stakeholder's insight about the effectiveness of the proposed interventions and the feasibility of implementing them.

## Conclusion

In Lebanon, challenges regarding MH were historically limited to vulnerable populations, such as migrant domestic workers and refugees. However, the devaluation of the Lebanese Lira, the COVID-19 pandemic, and the August 4th Beirut explosion have placed Lebanon in a seemingly endless state of crisis. The crises have decreased the Lebanese population's purchasing power, leaving women in the country with an unexpected dilemma: to spend their money on menstrual hygiene products or other necessities. Consequently, with the new onset of mass financial poverty, period poverty has spread throughout Lebanon as well. Women in Lebanon are resorting to unsafe practices that compromise their physical and mental health to a larger degree than ever before. To rectify this, three main challenges must be tackled: alleviating financial burden, protecting women's health, and fighting social stigma. It is necessary to take steps to address these issues to protect women's right to safe, clean, and accessible MH. While some measures must be implemented immediately, the fruits of many initiatives may not be seen for some time. While women in Lebanon navigate the turmoil of the ongoing crises, seeds can be sown today so that the newer generation is better equipped to handle gaps in women's health in general, including but not limited to MH.

## Data availability statement

The original contributions presented in the study are included in the article/supplementary material, further inquiries can be directed to the corresponding author/s.

## Ethics statement

Ethical review and approval was not required for the study on human participants in accordance with the local legislation and institutional requirements. Written informed consent for participation was not required for this study in accordance with the national legislation and the institutional requirements.

## Author contributions

All authors contributed to the data collection and write-up of this manuscript.

## Funding

FF is partly funded by the FCDO grant Rebuild for Resilience RBPS: 03873/ROC06913086JR.

## Conflict of interest

The authors declare that the research was conducted in the absence of any commercial or financial relationships that could be construed as a potential conflict of interest.

## Publisher's note

All claims expressed in this article are solely those of the authors and do not necessarily represent those of their affiliated organizations, or those of the publisher, the editors and the reviewers. Any product that may be evaluated in this article, or claim that may be made by its manufacturer, is not guaranteed or endorsed by the publisher.

## Abbreviations

MH: menstrual health
NGO: non-governmental organization
OBGYN: obstetrics and gynecology
MoPH: ministry of public health
UNEP: United Nations Environment Programme
UNFPA: United nations population fund
UN: United nations.
